# The impact of having foreign domestic workers on informal caregivers of persons with dementia – findings from a multi-method research in Singapore

**DOI:** 10.1186/s12877-022-03002-w

**Published:** 2022-04-08

**Authors:** Qi Yuan, Yunjue Zhang, Ellaisha Samari, Anitha Jeyagurunathan, Gregory Tee Hng Tan, Fiona Devi, Peizhi Wang, Harish Magadi, Richard Goveas, Li Ling Ng, Mythily Subramaniam

**Affiliations:** 1 Research Division, Institute of Mental Health, Buangkok Green, Medical Park, 10 Buangkok View, Singapore, 539747 Singapore; 2 School of Social Sciences, Singapore Management University, Singapore, Singapore; 3 Department of Geriatric Psychiatry, Institute of Mental Health, Singapore, Singapore; 4 Department of Psychological Medicine, Changi General Hospital, Singapore, Singapore

**Keywords:** Dementia, Informal caregiving, Foreign domestic helper, Propensity score matching, Qualitative research methods

## Abstract

**Background:**

Informal caregivers of persons with dementia (PWDs) sometimes engage foreign domestic workers (FDWs) to support their caregiving journey. However, there has not been much research to establish if this is really beneficial. The current study aims to investigate whether engaging FDWs specifically for caregiving of PWDs truly moderates caregiver stress and to explore caregivers’ experiences of engaging FDWs.

**Methods:**

A multi-method study design with a quantitative and qualitative sub-study was adopted. For the quantitative sub-study, 282 informal caregivers of PWDs were recruited. Propensity score matching analysis was used. For the qualitative sub-study, 15 informal caregivers with FDWs were interviewed. Inductive thematic analysis was conducted.

**Results:**

The quantitative sub-study confirmed that engaging FDWs did moderate the depressive symptoms of informal dementia caregivers (marginal effect = -3.35, *p* = 0.0497). However, such support did not affect their caregiving burden, self-efficacy, and perceived positive aspects of caregiving. The qualitative sub-study suggested that engaging FDWs is an ambivalent experience, which entails both support and challenges.

**Conclusions:**

The current study confirmed previous research findings, that engaging FDWs moderated depressive symptoms among caregivers of PWDs, and it could be through their physical support such as in daily caregiving activities. Policy-makers may consider providing more subsidies to caregivers caring for PWDs with mobility issues to hire FDWs. They may also consider providing training to FDWs on dementia caregiving skills and improving the intake of such training as this might be helpful for both FDWs and caregivers during this journey.

## Background

Dementia is a syndrome that is characterized by deterioration in memory, thinking, behavior, and the ability to perform daily activities. [1]. Such decline is usually progressive and irreversible [2, 3], and persons with dementia (PWDs) gradually lose their ability to live independently. As such, PWDs are often highly dependent on others and require significant assistance, especially during the middle and late stages of their illness [4]. Studies suggest that such care is mainly provided by family members of PWDs [5], and the informal caregiving process is often very stressful and demanding for caregivers [6, 7]. The high stress could be partly due to PWDs’ decline in cognition and function. Other possible reasons include the need for caregivers to support the personal care of PWDs, the isolation due to the long hours of caregiving, and the role conflict between being a caregiver versus responsibilities of other roles (e.g., being a parent or employee). As a result, caregivers of PWDs often report a high prevalence of depressive symptoms [8].

Home-based care is gaining more attention globally due to the much higher healthcare costs associated with institutional care [9]. However, due to societal changes including low birth rates, increasing longevity, and changing family structure, it’s getting more difficult for family members to provide such care. As a result, there is an increasing trend of hiring foreign domestic workers (FDWs) for this purpose in many places with developed economies [10]. This has been observed in Asia such as Taiwan [11] and Hong Kong [12], as well as in western societies, especially among some European countries [13–16]. In Singapore, this is a fairly common practice as previous studies have suggested that around half of the interviewed informal caregivers of PWDs reported that they had hired an FDW [2, 4, 17]. Despite the significant hiring of FDWs, less attention has been given to whether having an FDW is beneficial to caregivers of PWDs. There are some studies that have explored the impact of FDWs on the wellbeing of caregivers who take care of older adults and suggested the general positivity of such support to the informal caregivers [18, 19] and the older adults [20]. However, since the needs of older adults and PWD are quite different [21, 22], findings from studies on caregivers of older adults might not be applicable for those caring for PWDs. As such, studies are needed to address this gap.

The stress process model is a widely accepted model to understand caregiver stress [23]. This model proposes that the stress process is made up of four domains including the stress from background and context, the stressors, the mediators, and the outcomes. Particularly, the mediators here mainly refer to coping and social support. According to Pearline, these mediators might not only serve to lessen the intensity of stressors but also block the contagion between the primary and secondary stressors. This model might serve as the theoretical foundation to understand the impact of FDWs on caregivers. Major responsibilities for FDWs usually include domestic duties such as house chores and taking care of PWDs’ activity, as well as basic and instrumental activities of daily living [24]. Logically, such support should be able to reduce caregivers’ physical workload, lower the frequency of caregivers facing PWDs’ memory and behavior problems, and provide certain respite which can free caregivers to carry out other responsibilities. However, like two sides to a coin, introducing FDWs into the caregiving scenario results in caregivers both facing certain new challenges and receiving support from FDWs. To our knowledge, there is a dearth of literature that describes the full picture of engaging FDWs in the informal caregiving process, and this gap cannot be solved by a purely quantitative study. Although there was an earlier qualitative study that had explored the role of the FDWs for caregivers in Singapore, it was from the perspective of viewing FDWs as a form of support or coping resource [25].

Hence, the current study aims 1) to investigate if engaging FDWs to care for PWDs would moderate caregiver stress; and 2) to explore caregivers’ experiences of engaging FDWs. A multi-method research methodology was proposed to answer these two questions. Our hypotheses for the quantitative sub-study were as follows:

1. Informal caregivers taking care of PWDs who have engaged FDWs specifically to support caregiving would have lower depressive symptoms compared to those without FDWs;
2. Informal caregivers with FDWs would report less caregiving burden;
3. Informal caregivers with FDWs would have higher caregiving self-efficacy and perceived positive aspects of caregiving.

## Methods

### Quantitative sub-study

#### Participants and procedures

The quantitative sub-study is a cross-sectional survey of informal caregivers of PWDs in Singapore done from January 2017 to December 2018. The participants comprised a convenience sample of informal caregivers of PWDs who were mainly from the outpatient and satellite clinics of a tertiary psychiatric hospital in Singapore, the Institute of Mental Health, and a geriatric clinic in Changi General Hospital in Singapore. The study eligibility criteria were 1) Singapore residents (including citizens and permanent residents); 2) aged 21 years and above; 3) taking care of a patient who has been formally diagnosed with dementia; 4) and ability to communicate in either English, Mandarin or Malay. Caregivers who did not visit the PWDs every week or had difficulties in understanding the consent process were excluded. Since the main aim of this quantitative study was to explore the prevalence of depression among informal caregivers of PWD in Singapore, sample size was estimated based on the likely prevalence of depression in this sample. Based on the prevalence of 22.3% of depression from a systematic review [26], when alpha equals 0.05 and the precision is 0.05, the minimum sample size was estimated to be 267. A total of 282 informal caregivers were recruited at the end of this study, which was more than the minimum sample requirement. Data were collected via interviewer-administered questionnaires. Ethical approval for this study was obtained from the National Healthcare Group Domain Specific Review Board in Singapore (study reference number: 2016/00921). All participants provided written informed consent.

#### Measurements

PWDs’ cognitive impairment and functioning were reported by their caregivers. Functional dependence including activities of daily living and instrumental activities of daily living of PWDs were measured by the 6-item Activities of Daily Living Scale (ADL) [27] and the 8-item Instrumental Activities of Daily Living Scale (IADL) [28] separately. Memory and behaviour problems of PWDs were assessed by the memory (7 items) and behavior disruption (8 items) domains of the Revised Memory and Behaviour Problems Checklist (RMBPC) [29], and these two subscales were summed up as a single indicator of PWDs memory and behaviour problems.

Caregiver outcomes included 1) caregiving burden measured by the 22-item Zarit Burden Interview (ZBI) [30]; 2) caregiving self-efficacy assessed with the 15-item Revised Scale for Caregiving Self-Efficacy (RSCSE) [31]; 3) positive aspects of caregiving measured by the 9-item Positive Aspects of Caregiving Scale (PAC) [32]; and 4) depressive symptoms measured by the 20-item Centre for Epidemiologic Studies Depression Scale (CES-D) [33]. In a previous study, a 3-factor structure of 17 items was confirmed suitable for ZBI among the current population [34]; therefore this factor structure was used in this study. For the other scales, please refer to previously published studies for more details on their reliability and validity among the current sample [2, 34–36].

Socio-demographic information including caregiver’s age, gender, ethnicity, education level, marital status, and monthly household income was collected. Caregiving-related variables including relationship to the PWD, and whether caregivers engaged FDWs specifically for caregiving were also collected.

#### Analysis

To address the limitations of the traditional regression analysis, an alternative propensity score matching analysis was used in the current study, as this is a well-known method to estimate causal relationships in observational studies [37, 38]. The logic of this analysis is that each participant would be assigned with a single summary measure (i.e., propensity score) reflecting their odds of receiving the treatment based on observed background covariates [38]. Then one can match each treated individual to a comparator with a similar value of the propensity score. The advantage of this method is that it can rule out the effects of preexisting characteristics and make the interpretation of the findings more straightforward [39]. In the current study, treatment is defined as informal caregivers engaging FDWs specifically for caregiving. The measured covariates include caregivers’ demographics (age, gender, ethnicity, education level, and marital status), socioeconomic status (monthly household income), and caregiving-related variables (caregivers’ relationship with PWD and PWDs’ functional status). Multiple matching models including 1:1 nearest matching, 2:1 optimal ratio matching, and full matching were conducted, and the model with the best matching balance was selected for further analysis. The propensity score matching procedures were conducted via R package ‘Matchit’[40]. Standardized mean difference was used to assess if the covariates are balanced after the matching process [41], and a standardized mean difference of 0.1 or below indicates a good balance [42, 43]. As this threshold is more like a rule of thumb instead of a strict cutoff [37], a standardized mean difference of 0.2 or below is also considered acceptable following other previous studies [44, 45]. Since all the caregiver outcomes were continuous, we used weighted linear regression to assess the marginal effects of the treatment condition i.e., having engaged FDWs during the caregiving process in our study. All matching variables were still included in the regression model to control for small differences remaining in the matched samples after matching [46].

### Qualitative sub-study

#### Participants and procedures

The qualitative sub-study was a part of a larger qualitative study that was conducted between Apr 2019 to Dec 2020 to understand the caregiving experiences of local informal dementia caregivers. A convenience sampling was used to recruit the participants from the outpatient and satellite clinics of the Institute of Mental Health and a geriatric clinic in Changi General Hospital in Singapore. Additionally, the participants were also asked to refer their friends to join the study. The same inclusion and exclusion criteria as the quantitative sub-study were used except that Tamil-speaking caregivers were also included. Furthermore, caregivers of PWDs who were institutionalized in nursing homes at the point of recruitment were excluded. Data were collected via semi-structured interviews either through face-to-face conversation or via Zoom (after the outbreak of Covid-19). The final sample was determined by data saturation. In all, 29 caregivers were recruited for the parent project. Since the current study aimed to understand caregivers’ experiences of engaging FDWs in the informal caregiving process, those without FDWs were excluded. The final sample comprised 15 caregivers. This study was approved by the National Healthcare Group Domain Specific Review Board in Singapore (reference number: 2018/01069). Written informed consent was also obtained from all participants.

#### Analysis

All the interviews were audio-recorded and transcribed verbatim. The transcripts were checked by the facilitator first to ensure its consistency. The qualitative data was analyzed using the inductive thematic analysis approach [47], which allowed themes to emerge to answer the research question and enabled a low-level interpretation of the data [48]. Four random transcripts were distributed to the study team members (i.e., QY, YJZ, ES, and AJ), each independently reviewed the assigned transcript repeatedly to code meaningful data units. Then the team worked collaboratively to standardize, condense and group these data units into themes. A codebook with these themes, their definitions, inclusion and exclusion criteria, and typical examples was developed next to guide the coding process of all the remaining transcripts. This codebook was regularly refined till data saturation reached so there would be no new themes. Upon achieving a satisfactory kappa coefficient of 0.803, the remaining transcripts were distributed to the team members for independent coding. All the coding and analysis were conducted via NVivo 11 [49]. Minimal corrections such as grammar have been made to the verbatims presented in this study to ensure that proper English language is used.

## Results

The mean age of the participants (*n* = 282) in the quantitative sub-study was 55.7 ± 11.8, with a majority of them being female (75.2%), Chinese (83.0%), and were married/divorced/widowed (72.0%). Only 31.6% of them had an education level of degree or above, and around half of the family caregivers (52.1%) had a monthly household income of below SGD 5,999 (equivalent to USD 4,386). More than half of the caregivers were daughters of the PWDs (55.3%), followed by son-caregivers (17.0%) and spouse-caregivers (15.3%). Last but not least 43.6% of the informal caregivers reported that they received support from FDWs in caregiving. For the qualitative sub-study, the average age of the participants was 55.7 ± 4.6. The majority of the participants were female (80%), Chinese (86.7%), and daughter-caregivers (73.3%). Please refer to Table 1 for the details of sample characteristics of the two sub-studies.

**Table 1:** Characteristics of the study sample

**Variables**	**Quantitative sub-study**		**Qualitative sub-study**	
	**Frequency/Mean**	**Percentage/SD**	**Frequency/Mean**	**Percentage/SD**
**Independent/treatment variable**				
Caregiver receiving support from FDWs in taking care of PWDs				
Yes	123	43.6	-	-
No	159	56.4	-	-
**Matching variable**				
Age	55.7	11.8	55.7	4.6
Gender				
Male	70	24.8	3	20
Female	212	75.2	12	80
Ethnicity				
Chinese	234	83.0	13	86.7
Malay	29	10.3	1	6.7
Indian & others	19	6.7	1	6.7
Education level				
Secondary or below (include N/O level)	120	42.6	-	-
A level, polytechnic, and other diploma	73	25.9	-	-
Degree or above	89	31.6	-	-
Marital status				
Single	79	28.0	-	-
Married, divorced & widowed	203	72.0	-	-
Monthly household income				
< SGD5,999	147	52.1	-	-
SGD6,000 or above	89	31.6	-	-
NA/DK	46	16.3	-	-
Relationship to the PWD				
Spouse	43	15.3	-	-
Son	48	17.0	3	20
Daughter	156	55.3	11	73.3
Others	35	12.4	1	6.7
No. of PWD’s ADL	2.4	1.9	-	-
No. of PWD’s IADL	5.9	1.9	-	-
PWD’s memory and behaviour problems	6.9	3.1	-	-
**Outcome variables**				
CESD	14.3	11.0	-	-
ZBI (17 items)	23.6	13.4	-	-
impact on caregiver’s life	12.4	7.5	-	-
uncertainty over future	4.5	2.8	-	-
frustration	6.7	4.9	-	-
RSCSE—Obtain respite	65.9	29.0	-	-
RSCSE—Responding to disruptive behavior	65.2	24.0	-	-
RSCSE—Controlling upsetting thoughts	67.7	20.9	-	-
Positive aspects of caregiving	35.4	6.6	-	-

*SD* standard deviation

Table 2 shows the standardized mean differences of the covariates before and after the matching. Both model 1 and model 2 failed to meet our pre-defined criteria as the standardized mean differences of multiple covariates (e.g., ADL and IADL) were much higher than 0.2 after these two matching methods. For model 3 – full matching, the standardized mean differences of most covariates were below 0.1, with the standardized mean differences of only two variables which were slightly higher than 0.1 but still below 0.2, indicating adequate balance. In this case, the matched sample from model 3 was selected for further analysis. Since full matching utilized all the observations in the sample, none of the units were discarded.

**Table 2:** Standardized mean difference of controlled covariates before and after matching

**Variables**	**Pre-matched data**	**Model 1**	**Model 2**	**Model 3**
	**Std. Mean Diff**	**Std. Mean Diff**	**Std. Mean Diff**	**Std. Mean Diff**
Age	-0.016	-0.031	-0.030	-0.049
Gender				
Male	0.080	0.092	0.046	0.093
Female	-0.080	-0.092	-0.046	-0.093
Ethnicity				
Chinese	-0.076	0.021	-0.115	-0.081
Malay	0.017	-0.053	0.066	0.044
Indian & others	0.090	0.030	0.089	0.066
Education level				
Secondary or below (include N/O level)	-0.128	-0.167	-0.117	-0.115
A level, polytechnic, and other diploma	0.251	0.191	0.217	0.091
Degree or above	-0.122	-0.018	-0.099	0.030
Marital status				
Single	0.111	0.053	0.088	0.032
Married, divorced & widowed	-0.111	-0.053	-0.088	-0.032
Monthly household income				
< SGD5,999	-0.003	-0.016	0.049	0.075
SGD6,000 or above	0.036	0.069	-0.009	-0.007
NA/DK	-0.042	-0.068	-0.056	-0.094
Relationship to the PWD				
Spouse	-0.270	-0.212	-0.198	-0.042
Son	0.076	0.021	0.052	0.044
Daughter	-0.001	0.000	0.000	0.034
Others	0.149	0.158	0.113	-0.057
No. of PWD’s ADL	0.716	0.482	0.550	-0.076
No. of PWD’s IADL	1.188	0.495	0.963	-0.114
PWD’s memory and behaviour problems	-0.189	-0.150	-0.185	-0.033

Model 1 = 1:1 nearest matching, model = 2:1 optimal matching, and model 3 = full matching

Table 3 presents the marginal effects of regression analysis for caregivers’ outcomes if they had engaged FDWs specifically for caregiving using the weights generated from the full matching. After matching, support received from FDWs was associated with lower depression symptom scores of local informal dementia caregivers (marginal effect = -3.35, *p* = 0.0497). However, such support didn’t affect caregivers’ caregiving burden, self-efficacy, and perceived positive aspects of caregiving.

**Table 3:** Effects of receiving support for FDWs on caregivers’ outcomes after full matching

	Marginal effect	SE	t-value	*p*
CESD	-3.35	1.70	-1.97	**0.0497**
ZBI (17 items)	-0.91	1.65	-0.55	0.583
impact on caregiver’s life	-0.72	0.93	-0.78	0.439
uncertainty over future	0.49	0.36	1.35	0.178
frustration	-0.68	0.59	-1.16	0.246
RSCSE				
Obtain respite	-1.58	3.63	-0.43	0.664
Responding to disruptive behavior	1.16	3.64	0.32	0.750
Controlling upsetting thoughts	-0.04	3.45	-0.01	0.990
Positive aspects of caregiving	-0.43	0.81	-0.53	0.594

all analyses match on and adjust for all matching variables, *SE* standard error

Two major themes were identified from the qualitative sub-study, including support received by caregivers from FDWs and challenges related to FDWs encountered by caregivers. Two categories of support from FDWs were reported by informal caregivers: 1) support on daily caregiving tasks, and 2) emotional support. For challenges related to FDWs, the categories were: 1) challenges in finding and maintaining suitable FDWs; 2) FDWs’ lack of caregiving skills and proper attitudes; 3) training of FDWs; 4) challenges in managing the FDWs; 5) dependence on FDWs; and 6) dealing with FDWs’ personal issues.

### Support from FDWs

The support from FDWs was mainly on the daily caregiving tasks such as taking care of PWDs’ activities of daily living including bathing, dressing, toileting, transfer, continence, and feeding. Such support was more prominent if the PWD was immobile.

*‘Transferring is a difficult thing because she’s (PWD) totally on us. Because she cannot control herself, and her neck and everything. Luckily my helper was very, very well trained that she can lift her onto the bed independently and then we have to quickly use the hair dryer and dry her hair (PWD) you know, because her whole body is wet.’ – p05*

Other than physical support, caregivers also received emotional support from FDWs, and such emotional support, though much rarer, was helpful for the caregiving process as well.

*‘But my helper is good because she said that if your father knows that he has dementia, he will not behave in this manner. It’s because he doesn’t know, this is dementia she said. And she’s the one who taught us how to manage our temper.’–- P01*

### Challenges related to FDWs

Informal caregivers also encountered a lot of challenges in employing FDWs. The challenges could start from the process of finding suitable FDWs as the caregiving needs of different PWDs differ.

*‘But prior to that we had changed a few helpers because some of them […] they know that she is having dementia so they (FDWs) try to wriggle their way out with things that they want. So, when I discovered I was very angry. Then some (helpers were) a little bit more timid … then she (PWD) bullied people, she tried to hit people. And then some it’s like… just the chemistry is not there and my mum (PWD) just refused to respond.’ – p04*

Even if they were able to find a suitable FDW, another challenge expressed was in maintaining that FDW as there might be unforeseen circumstances.

*‘…This is the third helper we have […] the first one (helper) … her mother passed away, so she went back. My mom also was saying ‘maybe she won’t come back, so let her go and settle her things’. At first, she (FDW) said she wanted to stay and come back. But in the end, she decided she won’t. So, we said ‘ok, it’s fine.’ Then we had to move on…’ -p09*

At times, caregivers had to accept the FDWs available due to urgency as opposed to finding one who was well trained and more appropriate. But this lead to problems such as the FDWs not fitting caregivers’ expectations or FDWs lack of caregiving skills and proper attitudes, and caregivers need to train them.

*‘…that was very stressful […] I have to go to the office in the morning and then the doctor come around 8 o’clock, 9 o’clock […] so I have to catch the doctor to speak to him on what happened. Because the patient cannot communicate and the doctor also want to talk to me. It’s not possible to talk to my helper because she cannot convey the message and so it was a very difficult time that lasted the whole year. ‘-p05*

*‘For helpers, you want them to be happy and comfortable, but you also need to train and dementia is not easy to train you know. A lot of times people think they know dementia. But moderate level dementia is another thing because if they totally cannot talk and all that it’s one way of taking care. But my mom will still like to give orders. So, my helper has to figure out who to listen to and how […]. So, it was a headache, that part.’-p09*

Caregivers also encountered difficulties in managing the FDWs, for instance, when FDWs were less cooperative on the caregiving tasks.

*‘Sometimes I did scold my helper you know. Because she (FDW) kept on repeating the same mistake. I told her that it shouldn’t be like that but she would still repeat […] facing the dementia patient is already very stressful. Now facing another helper that don’t coordinate with you … stubborn… careless, you know. So it’s like I have burden plus burden …’ -p22*

However, caregivers’ reliance on FDWs’ support could create problems as caregivers may become too dependent on FDWs and be less capable of dealing with caregiving issues when FDWs are not around. A typical example is when the FDWs took leave, caregivers were unable to cope. They tend to look for alternative ways to ease the caregiving process and might need additional respite support.

*‘It was quite challenging because my helper has the day-off, sometimes once a month or twice a month. So, when she is off, we don’t cook. I will go and buy lunch and then buy dinner […] If one day my helper wants to go back or she wants to go back for home leave, then I think we need some physical support. Yah…otherwise, I would have to take leave or I would have to find respite care for my mum.’ – p03*

Last but not least, other than the caregiving-related difficulties related to PWDs, informal caregivers who choose to engage FDWs might also need to deal with FDWs’ personal or health issues.

*‘[…] after the first 2 years they (FDWs) have to go for medical examination right, the chest X-ray, my helper also. Her x-ray showed that there was a shadow in her lungs. Then the X-ray clinic called us to come back again [...] The result went back to her GP, and her GP said she had to go for a scan […]. The scan showed that she had a growth … mediastinal mass in her lungs […] and the growth was quite big […] she is a good helper and now she is sick, then I have to be her caregiver right, I mean that’s only fair. So, I told her, ‘Ok, I will find a surgeon here in Singapore to do it and I will settle my mom’ […] I don’t know why I never go into depression […]’ – p05*

## Discussion

It has been well established that caring for PWDs is stressful for informal caregivers, and this study highlights how engaging FDWs affects the caregiving journey. FDWs do provide support and practical assistance to caregivers, mainly through supporting daily caregiving tasks. Such support could moderate the depressive symptoms of caregivers. However, engaging FDWs is not without challenges, a theme that is unlike a previous study that purely focused on the positive impact of having FDWs [25]. Instead, it was an ambivalent experience which consisted of both positive and negative experiences. Previous studies have suggested similar ambivalent experiences between care recipients and caregivers [50], and between FDWs with care recipients (i.e. older adults) and employers (i.e., informal caregivers) [51, 52]. Our findings expand this ambivalent relationship, to informal caregivers of PWDs and their FDWs. As such, it might be inappropriate to view FDWs purely as a type of social support. This is different from studies that have examined the role of FDWs taking care of frail elderlies [19, 24]. Moreover, since this experience is mixed, it is possible that challenges related to FDWs might offset the support received by informal caregivers of PWDs. For future studies, researchers should treat FDWs both as a stressor and support, and to explore if one might affect the other to better understand their role in the stress process.

Findings from the quantitative sub-study suggested that engaging FDWs only moderated caregivers’ depressive symptoms, and it failed to improve the caregiving-related outcomes including caregiving burden, caregiving self-efficacy, and perceived positive aspects of caregiving. However, the qualitative sub-study suggested that FDWs do provide physical support such as assisting in daily caregiving activities or emotional support, similar to what was reported in previous studies [18, 25]. This is an interesting finding because intuitively, the direct impact of support from FDWs should be more on caregiving-related outcomes. There are several possible explanations for such a phenomenon. Firstly, unlike taking care of frail elderlies which mainly focus on the physical needs [24], a more typical problem for taking care of PWDs is that the caregivers need to deal with their memory and behavior problems and this was the single most important contributor to caregiving burden according to our previous study [34]. Engaging FDWs might reduce caregivers’ instances of facing such problems as the workload is shared by FDWs. However, this would not improve PWDs’ condition since dementia and its impact on PWDs is progressive and irreversible [2, 3]. Plausibly, for some of the more complex decisions besides assisting with ADLs, FDWs would still need to approach the primary caregiver for decisions. In other words, the decision-making responsibilities and the emotional burden still falls on the caregivers. As such the pressure faced by caregivers may not be reduced through engaging FDWs. Moreover, when FDWs get stressed or have burnout, caregivers would face dual stressors which might increase their perceived burden as they need to take care of both the PWDs and the stressed FDWs. Secondly, compared to caregiving-related outcomes, depressive symptoms are much more general and could be affected by stressors that are not caregiving specific. Though support from FDWs might not affect the mental stresses faced by caregivers, their support could be helpful for other stressors. For example, house chores—with the support from FDWs, caregivers can spend the time and energy which they had to spend on house chores previously on fulfilling other personal roles or responsibilities or even resting and this is very helpful for caregivers. Thirdly, the previous two explanations were both based on the assumptions that FDWs were cooperative and supportive, but these assumptions are not necessarily true at all times. When FDWs are not cooperative or supportive, indubitably, caregivers would face even more stress. For self-efficacy, the insignificance might be partly due to the wording of the questionnaire, as in the questionnaire respondents were asked to rate ‘how confident they are in finding a friend or family member’ in assisting them for various tasks. It’s possible some caregivers who did not treat FDW as their friend might not rate their confidence highly in this aspect [35]. Although support from FDWs may reduce caregivers’ frequency of facing the behavioral problems of PWDs, it will not improve their skills or abilities in managing these problems. As such, they may still lack the confidence of handling these problems. From this perspective, training caregivers on how to manage the behavioral problems of PWDs and how to manage upsetting thoughts would still be very helpful. Last but not the least, previous studies suggested that self-efficacy accounts for a significant proportion of PAC [53], and this might explain why engaging FDWs failed to improve the PAC of caregivers. Nevertheless, these are assumptions based on findings from our current multi-method study and previous studies; further research is needed to test these assumptions.

Findings from this study have practical implications. First of all, this study suggested that engaging FDWs is associated with a reduction of depressive symptoms among caregivers of PWDs. This might be particularly true if PWDs have mobility issues. As such, policymakers may consider providing more subsidies to families that are caring for immobile PWDs. Secondly, considering the global trend of home care for elderly [9, 54] (including Singapore [55, 56]), the policymakers should look into the needs of informal caregivers, regardless of those with or without FDWs as both groups require additional support just that the needs vary. Lastly, our study highlights the importance of providing training to FDWs on topics such as dementia-specific caregiving skills or language skills [24] and improving the intake rate of such training. This will benefit the FDWs as they will become more capable of handling the daily caregiving difficulties and as a result improve the caregiving journey for both FDWs and caregivers.

The current study has two major strengths. First of all, it is the first multi-method study that explored how engaging FDWs might affect caregivers of PWDs. Both quantitative and qualitative data were collected and presented. As a result, more comprehensive information was generated on this topic [57]. Secondly, it is also the first study to adopt a propensity score matching analysis strategy to test FDWs’ impact on caregivers of PWDs. The advantage of this methodology is that it tries to replicate a randomized experiment as closely as possible through obtaining treated and control groups with similar covariate distributions from observational data, therefore it is one of the most reliable methods for causal relationship inference among cross-sectional studies [58]. Therefore, the results are much more reliable compared to those from the traditional regression analysis strategies.

However, the following limitations must be kept in mind. Firstly, this study was conducted among informal dementia caregivers in Singapore and the participants were all self-selected, this might affect the generalizability of the study findings. Secondly, since data of both the quantitative and qualitative sub-studies were collected via interviews, social desirability bias might exist [59]. Lastly, our quantitative sub-study only had 282 participants which is considered to be a relatively small sample for propensity score matching. However, according to simulation studies, propensity score matching can still yield an accurate estimation of treatment effects even in small samples [60].

## Conclusion

Through a multi-method research design, the current study confirmed that FDWs could moderate the depressive symptoms among caregivers of PWDs mainly through providing physical support such as in daily caregiving tasks. This justifies the engagement of FDWs among caregivers to deal with their stress. Policy-makers might consider providing more subsidies to caregivers taking care of PWDs with mobility issues to hire FDWs. We also found that engaging FDWs was an ambivalent experience for caregivers which entailed both support and challenges. Service providers should consider training FDWs on topics such as dementia-specific caregiving skills or language skills as well as improving the intake rates of such training among FDWs, as this might improve the caregiving journey for both FDWs and caregivers.

## Data Availability

All individual data from this study resides with the Office of Research, Institute of Mental Health. Data is not available for online access, however, readers who wish to gain access to the data can write to the Clinical Research Committee, Institute of Mental Health/Woodbridge Hospital Secretariat at IMHRESEARCH@imh.com.sg. Access can be granted subject to the Institutional Review Board (IRB) and the research collaborative agreement guidelines. This is a requirement mandated for this research study by our IRB and funders.

## Abbreviations

PWD: Persons with dementia
FDW: Foreign domestic workers
ADL: Activities of Daily Living Scale
IADL: Instrumental Activities of Daily Living Scale
RMBPC: Revised Memory and Behaviour Problems Checklist
ZBI: Zarit Burden Interview
RSCSE: Revised Scale for Caregiving Self-Efficacy
PAC: Positive Aspects of Caregiving Scale
CES-D: Centre for Epidemiologic Studies Depression Scale

## References

[1] Dementia [https://www.who.int/news-room/fact-sheets/detail/dementia]

[2] Yuan Q, Wang P, Tan TH, Devi F, Poremski D, Magadi H, Goveas R, Ng LL, Chong SA, Subramaniam M. Coping patterns among primary informal dementia caregivers in Singapore and its impact on caregivers—Implications of a latent class analysis. The Gerontologist. 2020;61(5):680–92. 10.1093/geront/gnaa080 PMC8276612 32592582

[3] Harris D (2007). Forget me not: palliative care for people with dementia. Postgrad Med J.

[4] Yuan Q, Tan TH, Wang P, Devi F, Ong HL, Abdin E, Harish M, Goveas R, Ng LL, Chong SA (2020). Staging dementia based on caregiver reported patient symptoms: Implications from a latent class analysis. PLOS ONE.

[5] Ciccarelli N, Van Soest A (2018). Informal caregiving, employment status and work hours of the 50+ population in Europe. De Economist.

[6] Butcher HK, Holkup PA, Buckwalter KC (2001). The experience of caring for a family member with Alzheimer’s disease. West J Nurs Res.

[7] Brodaty H, Donkin M (2009). Family caregivers of people with dementia. Dialogues Clin Neurosci.

[8] Sallim AB, Sayampanathan AA, Cuttilan A (2015). Ho RC-M: Prevalence of mental health disorders among caregivers of patients with Alzheimer disease. J Am Med Dir Assoc.

[9] Fisher O (2021). The Impact of Micro and Macro Level Factors on the Working and Living Conditions of Migrant Care Workers in Italy and Israel-A Scoping Review. Int J Environ Res Public Health.

[10] Ho KHM, Chiang VCL, Leung D, Cheung DSK (2019). A feminist phenomenology on the emotional labor and morality of live-in migrant care workers caring for older people in the community. BMC Geriatr.

[11] Peng LM, Chiu YC, Liang J, Chang TH (2018). Risky wandering behaviors of persons with dementia predict family caregivers’ health outcomes. Aging Ment Health.

[12] Chong AML, Kwan CW, Lou VWQ, Chi I (2017). Can domestic helpers moderate distress of offspring caregivers of cognitively impaired older adults?. Aging Ment Health.

[13] Österle A, Bauer G (2012). Home care in Austria: the interplay of family orientation, cash-for-care and migrant care. Health Soc Care Community.

[14] Horn V, Schweppe C, Böcker A, Bruquetas Callejo M. Live-in migrant care worker arrangements in Germany and the Netherlands. Motivations and justifications in family decision-making. Int J Ageing Later Life. 2019;13(2):83–113.

[15] Barbabella F, Chiatti C, Rimland JM, Melchiorre MG, Lamura G, Lattanzio F (2016). Socioeconomic Predictors of the Employment of Migrant Care Workers by Italian Families Assisting Older Alzheimer’s Disease Patients: Evidence From the Up-Tech Study. J Gerontol B Psychol Sci Soc Sci.

[16] Romero BA (2012). Towards a model of externalisation and denationalisation of care? The role of female migrant care workers for dependent older people in Spain. Eur J Soc Work.

[17] Tew CW, Tan LF, Luo N, Ng WY, Yap P (2010). Why Family Caregivers Choose to Institutionalize a Loved One with Dementia: A Singapore Perspective. Dement Geriatr Cogn Disord.

[18] Chong AML, Kwan CW, Chi I, Lou VWQ, Leung AYM (2014). Domestic Helpers as Moderators of Spousal Caregiver Distress. J Gerontol: Series B.

[19] Østbye T, Malhotra R, Malhotra C, Arambepola C, Chan A (2013). Does support from foreign domestic workers decrease the negative impact of informal caregiving? Results from Singapore survey on informal caregiving. J Gerontol B Psychol Sci Soc Sci.

[20] Ho KHM, Cheung DSK, Lee PH, Lam SC, Kwan RYC. Co-living with migrant domestic workers is associated with a lower level of loneliness among community-dwelling older adults: A cross-sectional study. 10.1111/hsc.13520 34288198

[21] Abdi S, Spann A, Borilovic J, de Witte L, Hawley M (2019). Understanding the care and support needs of older people: a scoping review and categorisation using the WHO international classification of functioning, disability and health framework (ICF). BMC Geriatr.

[22] Whitlatch CJ, Orsulic-Jeras S (2018). Meeting the Informational, Educational, and Psychosocial Support Needs of Persons Living With Dementia and Their Family Caregivers. Gerontologist.

[23] Pearlin LI, Mullan JT, Semple SJ, Skaff MM (1990). Caregiving and the stress process: An overview of concepts and their measures. Gerontologist.

[24] Ha NHL, Chong MS, Choo RWM, Tam WJ, Yap PLK (2018). Caregiving burden in foreign domestic workers caring for frail older adults in Singapore. Int Psychogeriatr.

[25] Basnyat I, Chang L (2017). Examining Live-In Foreign Domestic Helpers as a Coping Resource for Family Caregivers of People With Dementia in Singapore. Health Commun.

[26] Cuijpers P (2005). Depressive disorders in caregivers of dementia patients: a systematic review. Aging Ment Health.

[27] Katz S, Ford AB, Moskowitz RW, Jackson BA, Jaffe MW (1963). Studies of illness in the aged: the index of ADL: a standardized measure of biological and psychosocial function. JAMA.

[28] Lawton MP, Brody EM (1969). Assessment of Older People: Self-Maintaining and Instrumental Activities of Daily Living. The Gerontologist.

[29] Teri L, Truax P, Logsdon R, Uomoto J, Zarit S, Vitaliano PP (1992). Assessment of behavioral problems in dementia: the revised memory and behavior problems checklist. Psychol Aging.

[30] Zarit SH, Reever KE, Bach-Peterson J (1980). Relatives of the impaired elderly: correlates of feelings of burden. Gerontologist.

[31] Steffen AM, McKibbin C, Zeiss AM, Gallagher-Thompson D, Bandura A (2002). The revised scale for caregiving self-efficacy: reliability and validity studies. J Gerontol B Psychol Sci Soc Sci.

[32] Tarlow BJ, Wisniewski SR, Belle SH, Rubert M, Ory MG, Gallagher-Thompson D (2004). Positive Aspects of Caregiving: Contributions of the REACH Project to the Development of New Measures for Alzheimer’s Caregiving. Res Aging.

[33] Radloff LS (1977). The CES-D scale a self-report depression scale for research in the general population. Appl Psychol Meas.

[34] Yuan Q, Tan GTH, Wang P, Devi F, Goveas R, Magadi H, Ng LL, Chong SA, Subramaniam M (2021). Combining a variable-centered and a person-centered analytical approach to caregiving burden – a holistic approach. BMC Geriatr.

[35] Tan GTH, Yuan Q, Devi F, Wang P, Ng LL, Goveas R, Chong SA, Subramaniam M (2021). Factors associated with caregiving self-efficacy among primary informal caregivers of persons with dementia in Singapore. BMC Geriatr.

[36] Devi F, Yuan Q, Wang P, Tan GTH, Roshan Goveas R, Ng LL, Chong SA, Subramaniam M (2020). Positive aspect of caregiving among primary informal dementia caregivers in Singapore. PLOS ONE.

[37] Harder VS, Stuart EA, Anthony JC (2010). Propensity score techniques and the assessment of measured covariate balance to test causal associations in psychological research. Psychol Methods.

[38] Stuart EA, Green KM (2008). Using full matching to estimate causal effects in nonexperimental studies: examining the relationship between adolescent marijuana use and adult outcomes. Dev Psychol.

[39] Thibodeau MA, Welch PG, Sareen J, Asmundson GJ (2013). Anxiety disorders are independently associated with suicide ideation and attempts: propensity score matching in two epidemiological samples. Depress Anxiety.

[40] Ho D, Imai K, King G, Stuart EA. MatchIt: Nonparametric Preprocessing for Parametric Causal Inference. J Stat Softw. 2011;42(8):1–28.

[41] Austin PC (2011). An Introduction to Propensity Score Methods for Reducing the Effects of Confounding in Observational Studies. Multivar Behav Res.

[42] Normand ST, Landrum MB, Guadagnoli E, Ayanian JZ, Ryan TJ, Cleary PD, McNeil BJ (2001). Validating recommendations for coronary angiography following acute myocardial infarction in the elderly: a matched analysis using propensity scores. J Clin Epidemiol.

[43] Zhang Z, Kim HJ, Lonjon G, Zhu Y (2019). Balance diagnostics after propensity score matching. Ann Transl Med.

[44] McCaffrey DF, Griffin BA, Almirall D, Slaughter ME, Ramchand R, Burgette LF (2013). A tutorial on propensity score estimation for multiple treatments using generalized boosted models. Stat Med.

[45] Tein JY, Mazza GL, Gunn HJ, Kim H, Stuart EA, Sandler IN, Wolchik SA (2018). Multigroup Propensity Score Approach to Evaluating an Effectiveness Trial of the New Beginnings Program. Eval Health Prof.

[46] Ho DE, Imai K, King G, Stuart EA (2007). Matching as nonparametric preprocessing for reducing model dependence in parametric causal inference. Polit Anal.

[47] Braun V, Clarke V (2006). Using thematic analysis in psychology. Qual Res Psychol.

[48] Vaismoradi M, Turunen H, Bondas T (2013). Content analysis and thematic analysis: Implications for conducting a qualitative descriptive study. Nurs Health Sci.

[49] QSR International (2015). NVivo qualitative data analysis [Computer software].

[50] Iecovich E (2014). The Association Between Older Israelis’ Quality of Relationships With Their Family and Migrant Live-in Caregivers and Their Loneliness. J Gerontol: Series B.

[51] Ayalon L (2009). Family and family-like interactions in households with round-the-clock paid foreign carers in Israel. Ageing Soc.

[52] Walsh K, Shutes I (2013). Care relationships, quality of care and migrant workers caring for older people. Ageing Soc.

[53] Semiatin AM, O’Connor MK (2012). The relationship between self-efficacy and positive aspects of caregiving in Alzheimer’s disease caregivers. Aging Ment Health.

[54] Ris I, Schnepp W, Mahrer Imhof R (2019). An integrative review on family caregivers’ involvement in care of home-dwelling elderly. Health Soc Care Community.

[55] Yeoh BS, Huang S (2010). Foreign domestic workers and home-based care for elders in Singapore. J Aging Soc Policy.

[56] Successful Ageing — A Review of Singapore’s Policy Approaches [https://www.csc.gov.sg/articles/successful-ageing-a-review-of-singapore%27s-policy-approaches]

[57] Hammond C (2005). The Wider Benefits of Adult Learning: An Illustration of the Advantages of Multi-method Research. Int J Soc Res Methodol.

[58] Stuart EA (2010). Matching methods for causal inference: A review and a look forward. Stat Sci.

[59] Bowling A (2005). Mode of questionnaire administration can have serious effects on data quality. J Public Health.

[60] Pirracchio R, Resche-Rigon M, Chevret S (2012). Evaluation of the Propensity score methods for estimating marginal odds ratios in case of small sample size. BMC Med Res Methodol.

